# Occupational hazard incident reporting systems in health facilities across sub-Saharan Africa: A scoping review protocol

**DOI:** 10.1371/journal.pone.0360189

**Published:** 2026-10-06

**Authors:** Innocent Ayesiga, David Tenywa, Moses Ntaro

**Affiliations:** 1 Department of Community Health, Mbarara University of Science and Technology, Mbarara, Uganda; 2 Department of Laboratory, Ruharo Mission Hospital, Mbarara, Uganda; Zydus Research Center, INDIA

## Abstract

**Introduction:**

Occupational hazards (OH) in healthcare facilities are common, with 1 in 10 healthcare workers experiencing a hazard each year. These hazards can be either biological, chemical, ergonomic, or radiation. Despite their occurrence, reporting of these hazards remains limited, with very little information available, especially from healthcare facilities. Also, there is limited information on incident reporting systems (IRSs), which can help capture, track, and monitor the occurrence of these hazards within health facilities. The absence of these systems results in inaccurate and incomplete reporting and fragmented data, hindering the implementation of effective occupational safety measures in hospitals. This review will explore the extent and nature of information available on occupational hazard incident reporting systems in Sub-Saharan Africa.

**Methods:**

The review will be conducted using the Joanna Briggs Institute (JBI) methodology of scoping reviews and reported in accordance with the Preferred Reporting Items for Systematic Reviews and Meta-Analyses Extension for Scoping Reviews (PRISMA-ScR). A robust search strategy shall be conducted in different scientific databases, including PubMed, Embase, Scopus, and Web of Science, guided by the Population, Concept, and Context framework. Studies describing incident reporting systems shall be considered in this review. The search results will be collated and uploaded to Covidence, where duplicates will be removed and titles and abstracts screened. Search and screening of studies have not yet commenced.

**Discussion and conclusion:**

Occupational health and safety are vital to the functioning of healthcare facilities because they promote well-being and protect both patients and healthcare workers. It is vital to ensure accurate and robust IRS are in place, especially given the occurrence of occupational hazards across hospital wards. These IRS will help with surveillance and the identification of epidemiological trends in OH among health workers.

**Protocol Registration:**

The protocol has not been registered.

## Introduction

Globally, occupational hazards (OH) in healthcare facilities continue to hinder effective service delivery by health workers. These occupational hazards may include physical, chemical, ergonomic, radiation, biological, and electrical hazards in hospital settings [1]. Healthcare workers are also increasingly exposed to psychosocial occupational hazards that substantially affect their quality of life and well-being [2]. These include occupational stress, burnout, excessive workload, shift work, fatigue, workplace bullying, moral distress, verbal abuse, sexual harassment, and physical violence from patients. Estimates indicate that 1 in 10 health workers globally experience an occupational hazard each year [3]. Reviews from Sub-Saharan Africa (SSA) have reported varying prevalences of occupational hazards; for example, Mossburg et al. reported a prevalence proportion of 22–95% [3]. A scoping review conducted in Tanzania reported a prevalence ranging from 27.1% to 59.2% across 6 studies, while another scoping review across Low- and middle-income countries (LMICs) reported a prevalence ranging from 27% to 82% across 12 studies [4,5]. Other reviews have evaluated the presence of incident reporting systems for patient safety, which assess practices aimed at ensuring patients receive optimal care [6]. These reviews demonstrate the prevalence of the problem across the continent. However, despite classification of occupational hazards, their reporting remains limited due to the lack of robust infrastructure and reporting systems. Yet effective occupational health and safety (OHS) systems within health facilities rely heavily on robust incident-reporting mechanisms to identify risks, monitor trends, and guide prevention strategies.

Occupational hazard Incident reporting systems are platforms that help track and monitor incidents within the health facility. These incident reporting systems (IRS) can be used by both health workers and patients to capture incidents [7]. This reporting enables early characterisation of the incident, its level of risk, and its impact on individuals across the health facility. These systems also facilitate the timely response to the hazard, ensure appropriate post-exposure management, and enable effective surveillance [8]. The IRS also contribute to the development of a safety culture, accountability, and continuous quality improvement. These reporting systems can either be paper-based or electronic at either primary, secondary, or tertiary healthcare levels. They can also be applied in different ward settings, such as surgery, medical, paediatrics, or outpatient wards [8]. OH IRSs have been implemented in various developed countries, including Scotland, Spain, Italy, the Netherlands, and Canada [9–13].

Many facilities across SSA often operate in resource-constrained settings characterised by high patient volumes, limited personnel, and inadequate personal protective equipment (PPE) [14,15]. These challenges increase both the risk of occupational hazards and the likelihood of underreporting incidents. Evidence from facility-based studies suggests that occupational incidents are often underreported [16]. For example, Bahat et al. demonstrated approximately 46% underreporting of needlestick injuries among 2205 Health workers [17]. Most of this underreporting arises from unclear reporting procedures, the absence of reporting systems, fear of blame, a lack of confidentiality, a lack of feedback, and limited institutional support for occupational health. This underreporting has resulted in very limited, fragmented information on incidence, prevalence, and hazard-specific data across different health facilities. The absence of this information hinders the development of effective occupational hazard and safety strategies to counteract their occurrence.

Additionally, effective occupational hazard incident reporting systems have important implications for nursing education, clinical training, and workforce development. Information generated through these systems provides valuable learning opportunities for students, nurses, physicians, and allied health professionals. This information can identify recurring workplace hazards and inform safety training programmes. It can also strengthen risk communication and promote a culture of patient and worker safety. Incident reporting data can guide curriculum development for continuing professional education and simulation-based learning. These learning processes lead to institutional quality improvement initiatives that can reduce occupational injuries among frontline healthcare workers. Current reviews conducted on the continent have prioritised examining the prevalence and types of occupational hazards among healthcare workers [3–5]. Therefore, we intend to explore what is known and how much is known about occupational hazard incident reporting systems in health facilities in SSA. We believe this review will identify gaps in policy, implementation, and research related to the IRS to improve OHS in health facilities.

## Methods

This scoping review shall be informed by the Joanna Briggs Institute (JBI) methodology [18]. The review will be reported in accordance with the Preferred Reporting Items for Systematic Reviews and Meta-Analyses Extension for Scoping Reviews (PRISMA-ScR). A scoping review has been chosen to explore current data on integrating IRSs within healthcare facilities in SSA. The population-concept-context (PCC) framework will guide the development of eligibility criteria. The population shall include all health facilities, both public and private, that implement incident hazard reporting systems. These facilities shall also be considered at both primary, secondary, and tertiary care levels within SSA. The review shall examine the various attributes of occupational hazard incident reporting systems, including the mechanisms, tools, policies, processes, and practices involved in reporting. Context will prioritise the health facilities within SSA.

### Primary research question

What evidence exists on the occupational hazard incident reporting systems used in Hospitals in SSA?

### Secondary research questions

1. What type of occupational hazard incident reporting systems are being implemented in health facilities?
2. How are reporting systems structured and operationalised within health facilities in SSA?
3. What is known about the utilisation, completeness, and effectiveness of these reporting systems at the facility level?
4. What institutional, organisational, and contextual barriers and facilitators influence incident reporting in health facilities across SSA?
5. What gaps exist in policy, implementation, and research related to occupational hazard reporting systems in health facilities across SSA?

### Identifying relevant studies

A comprehensive search strategy shall be developed following consultation with a librarian. Various electronic databases, including PubMed/MEDLINE, Scopus, Web of Science, Embase, African Journals Online and CINAHL, will be used in the search strategy. Grey literature shall also be considered, and a search will be conducted across databases, including the World Health Organisation, the International Labour Organisation, Ministries of Health Websites, conference proceedings, and reports from theses and dissertation databases. The search will include studies published between 1990 and the date of the final search. No language restrictions will be applied, although studies not published in English will be translated where feasible. A combination of Medical Subject Headings (MeSH) and free-text terms will be used, including terms related to occupational hazards, incident reporting, surveillance systems, health workers, and Sub-Saharan Africa, as demonstrated in Table 1. Both controlled vocabularies, like MeSH and CINAHL Headings, and free-text keywords will be used. Boolean operators, OR and AND, shall be used to link the search strings.

**Table 1:** Search Strings for the different databases.

Database	Search String
PubMed	(“Occupational Exposure”[Mesh] OR “Occupational Injuries”[Mesh] OR “Needlestick Injuries”[Mesh] OR “Accidents, Occupational”[Mesh] OR “Workplace Violence”[Mesh] OR occupational hazard* OR occupational exposure* OR occupational injur* OR workplace hazard* OR workplace injur* OR needlestick injur* OR sharps injur* OR blood exposure* OR biological exposure*) **AND** (“Incident Reporting”[Mesh] OR “Safety Management”[Mesh] OR “Disease Notification”[Mesh] OR reporting system* OR incident report* OR incident reporting OR adverse event reporting OR occupational incident reporting OR safety reporting system* OR surveillance system* OR reporting mechanism* OR reporting practice*) **AND** (“Health Facilities”[Mesh] OR “Hospitals”[Mesh] OR “Primary Health Care”[Mesh] OR “Health Personnel”[Mesh] OR health facilit* OR hospital* OR healthcare setting* OR healthcare facilit* OR clinic* OR medical center* OR health institution*) **AND** (“Africa South of the Sahara”[Mesh] OR “Sub-Saharan Africa” OR “SSA” OR Angola OR Benin OR Botswana OR “Burkina Faso” OR Burundi OR Cameroon OR “Cape Verde” OR “Central African Republic” OR Chad OR Comoros OR Congo OR “Democratic Republic of Congo” OR Djibouti OR Eritrea OR Eswatini OR Ethiopia OR Gabon OR Gambia OR Ghana OR Guinea OR “Guinea-Bissau” OR Kenya OR Lesotho OR Liberia OR Madagascar OR Malawi OR Mali OR Mauritania OR Mauritius OR Mozambique OR Namibia OR Niger OR Nigeria OR Rwanda OR “Sao Tome and Principe” OR Senegal OR Seychelles OR “Sierra Leone” OR Somalia OR “South Africa” OR “South Sudan” OR Sudan OR Tanzania OR Togo OR Uganda OR Zambia OR Zimbabwe)
EMBASE	(’occupational exposure’/exp OR ‘occupational injury’/exp OR ‘needlestick injury’/exp OR ‘workplace violence’/exp OR occupational hazard*:ti,ab OR occupational exposure*:ti,ab OR occupational injur*:ti,ab OR workplace injur*:ti,ab OR needlestick injur*:ti,ab OR sharps injur*:ti,ab) **AND** (’incident reporting’/exp OR ‘safety management’/exp OR ‘disease surveillance’/exp OR incident report*:ti,ab OR incident reporting:ti,ab OR safety reporting system*:ti,ab OR occupational incident reporting:ti,ab OR surveillance system*:ti,ab OR reporting mechanism*:ti,ab) **AND** (’health care facility’/exp OR ‘hospital’/exp OR ‘primary health care’/exp OR health facilit*:ti,ab OR hospital*:ti,ab OR healthcare setting*:ti,ab OR clinic*:ti,ab) **AND** (’sub-saharan africa’/exp OR ‘africa south of the sahara’:ti,ab OR ‘sub-saharan africa’:ti,ab OR Angola OR Benin OR Botswana OR ‘Burkina Faso’ OR Burundi OR Cameroon OR ‘Central African Republic’ OR Chad OR Congo OR ‘Democratic Republic of Congo’ OR Ethiopia OR Ghana OR Kenya OR Malawi OR Mozambique OR Namibia OR Nigeria OR Rwanda OR Senegal OR Somalia OR ‘South Africa’ OR Tanzania OR Uganda OR Zambia OR Zimbabwe)
Scopus	TITLE-ABS-KEY (“occupational hazard*” OR “occupational exposure*” OR “occupational injur*” OR “workplace injur*” OR “needlestick injur*” OR “sharps injur*” OR “blood exposure*” OR “biological exposure*”) **AND** (“incident reporting” OR “incident report*” OR “occupational incident reporting” OR “safety reporting system*” OR “incident surveillance” OR “reporting mechanism*” OR “adverse event reporting”) **AND** (“health facilit*” OR hospital* OR “healthcare setting*” OR clinic* OR “medical center*”) **AND** (“sub-saharan africa” OR “africa south of the sahara” OR Angola OR Benin OR Botswana OR “Burkina Faso” OR Burundi OR Cameroon OR “Central African Republic” OR Chad OR Congo OR “Democratic Republic of Congo” OR Ethiopia OR Ghana OR Kenya OR Malawi OR Mozambique OR Namibia OR Nigeria OR Rwanda OR Senegal OR “South Africa” OR Tanzania OR Uganda OR Zambia OR Zimbabwe))
CINAHL	(MH “Occupational Exposure+” OR MH “Occupational Injuries+” OR MH “Needlestick Injuries+” OR occupational hazard* OR occupational exposure* OR occupational injur* OR workplace injur* OR needlestick injur* OR sharps injur*) **AND** (MH “Incident Reporting+” OR MH “Safety Management+” OR incident reporting OR incident report* OR adverse event reporting OR safety reporting system* OR occupational incident reporting OR reporting mechanism* OR surveillance system*) **AND** (MH “Health Facilities+” OR MH “Hospitals+” OR MH “Primary Health Care+” OR health facilit* OR hospital* OR healthcare setting* OR clinic*) **AND** (MH “Africa South of the Sahara+” OR “Sub-Saharan Africa” OR Angola OR Benin OR Botswana OR “Burkina Faso” OR Burundi OR Cameroon OR Chad OR Congo OR Ethiopia OR Ghana OR Kenya OR Malawi OR Mozambique OR Namibia OR Nigeria OR Rwanda OR Senegal OR “South Africa” OR Tanzania OR Uganda OR Zambia OR Zimbabwe)

### Study selection

All the identified records will be uploaded to Covidence, an online screening tool [18]. Two reviewers will independently screen titles and abstracts against the inclusion criteria. The included studies will be evaluated and independently reviewed as full texts by the same reviewers. Any discrepancies at any stage will be resolved by a third reviewer. The screening and study selection process will be document and illustrations will be made using the PRISMA-ScR flow diagram as demonstrated in Fig 1 [19].

**Fig 1:**
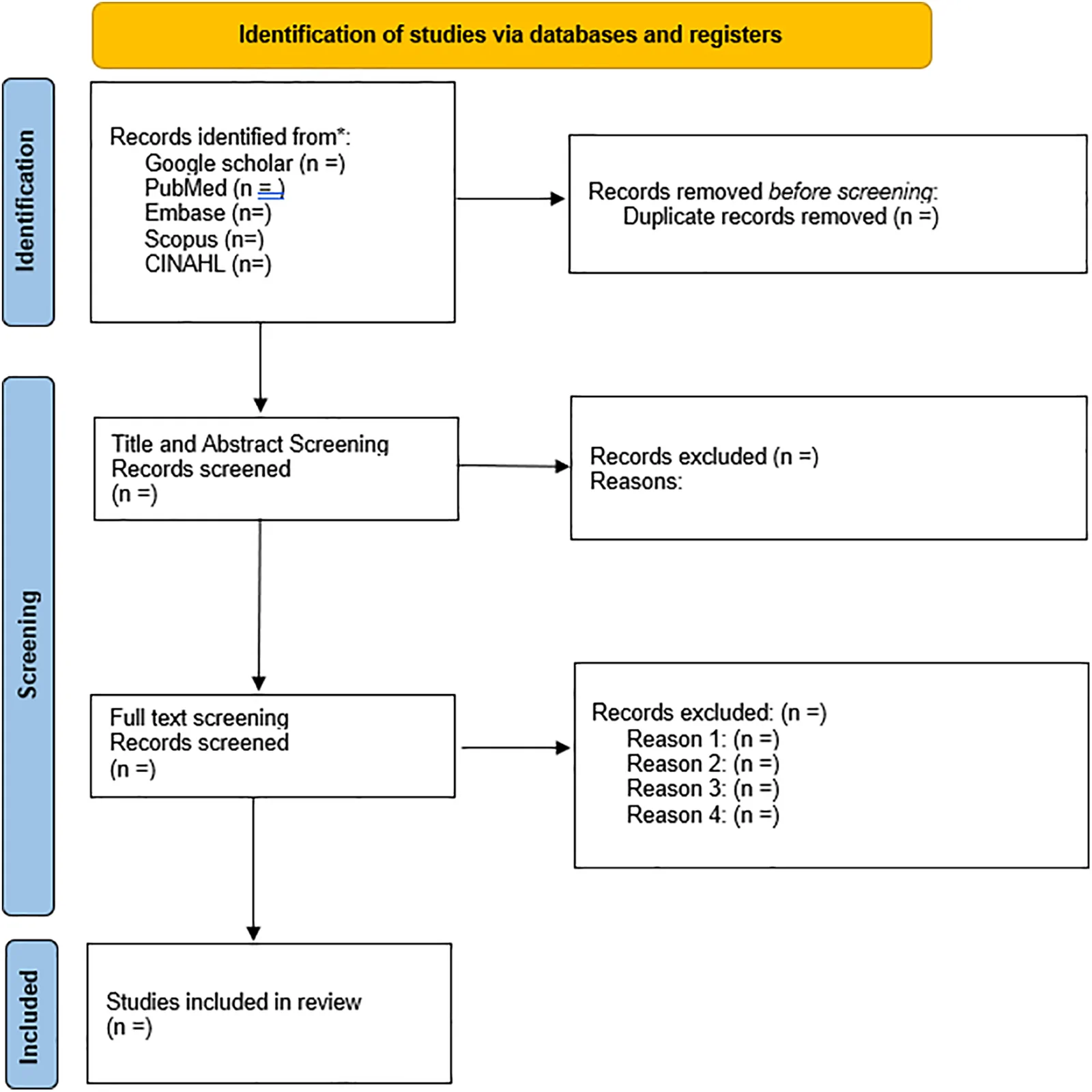
Flow diagram of how the studies shall be identified, screened, and included.

### Inclusion criteria

Studies that shall be included in this review will be evaluated against the criteria below.

- They must be conducted in health facilities in SSA.
- Studies must describe, evaluate, and discuss occupational hazard incident reporting systems or mechanisms at the facility level.
- Quantitative, qualitative, mixed, or quasi-experimental studies will be included.

### Exclusion criteria

Studies will be excluded if they meet the following criteria.

- All other reviews, including narrative, scoping, systematic, state-of-the-art, and umbrella reviews.
- Studies conducted outside SSA.
- Modelling studies without reports on incident reporting systems.
- Studies solely focusing on the prevalence of occupational hazards without reporting an incident reporting system.
- Studies reporting on the knowledge, attitudes, and practices about occupational hazards, but not reporting on incident reporting systems.

### Data extraction and reporting

Data will be charted using a data extraction tool that captures parameters such as Author(s), year of publication, and country. Other details to be extracted will include study design, target population, study objectives, characteristics of the incident reporting system, health worker cadres, name of reporting system, country and health facility level, type of reporting system (paper-based, electronic, hybrid), governance/administrative structure, purpose and objectives of the system, hazard categories captured (biological, chemical, physical, ergonomic, psychosocial), reporting mechanisms, reporting frequency, reporting population, data elements collected, feedback mechanisms, data analysis approaches, integration with Occupational Safety and Health programs, integration with national health information systems, confidentiality and anonymity provisions, reporting thresholds, indicators monitored, utilization of collected information, and system outcomes. Where available, information describing system performance and implementation challenges will also be extracted, including underreporting, reporting barriers, fear of blame, resource limitations, technological constraints, lack of staff awareness, inadequate management support, poor feedback mechanisms, and sustainability challenges. Results will be presented in tables, charts, and narrative summaries. Where appropriate, the results will be organised thematically to highlight the common patterns and contextual differences across SSA. Data extraction will be performed independently by two individuals to ensure that adequate information is extracted.

### Critical appraisal

An assessment of the methodological quality of included studies will be undertaken to provide additional context for interpreting the mapped evidence. The appraisal will not be used as a basis for excluding studies but rather to describe the methodological strengths and limitations of the available literature and identify areas where higher-quality evidence is needed. The methodological quality of empirical studies will be assessed independently by two reviewers using either the Mixed Methods Appraisal Tool (MMAT) 2018 or the Joanna Briggs Institute (JBI) Critical Appraisal Checklist [20,21]. These checklists will be selected according to the study design, for example, cross-sectional, qualitative, cohort, case-control, or mixed-methods. MMAT will be used because it allows appraisal of qualitative, quantitative, and mixed-methods studies within a single framework [21]. Any disagreements between reviewers will be resolved through discussion, and where consensus cannot be reached, a third reviewer will arbitrate. The results of the quality appraisal will be summarised narratively and presented in tabular form to complement the scoping review’s findings.

### Current status of the study

The review has not yet commenced.

### Checklist

A PRISMA-ScR protocol checklist is included as S1 File [22].

## Discussion and conclusion

OHS is a vital component in improving the functionality of health systems, motivating HWs, and ensuring the safety of both patients and HWs. Occupational Hazards are common occurrences across different health facilities, and these put the lives of both patients and HWs at risk of infection and disability [23]. The IRS is a vital component within health facilities for tracking, monitoring, and identifying occupational hazards. Establishing IRS is also critical to enhancing data capture, which is essential for promoting evidence-based decision-making. However, occupational hazard incident reporting systems have different limitations despite their relevance in improving workplace safety. These systems are largely reactive in nature and rely on incidents occurring before risks are identified [24]. They have limited ability to proactively identify emerging hazards or prevent future incidents. Incident reporting systems frequently capture only a fraction of actual occupational hazards because of substantial underreporting. Reporting may be discouraged by multiple factors, such as fear of blame, disciplinary action, litigation, perceived lack of confidentiality, heavy workloads, reporting fatigue, or the belief that reporting will not result in meaningful organizational change [25]. Additional limitations include inconsistent reporting practices and variable data quality. These weaknesses may reduce the usefulness of incident reporting data for surveillance and prevention planning.

Occupational hazard incident reporting systems should be considered one component of a comprehensive occupational health surveillance strategy. Occupational Safety and Health (OSH) Monitoring Systems adopt a broader preventive approach by continuously monitoring workplace risks and safety practices. They also monitor compliance with occupational safety standards and the implementation of preventive interventions. These complementary systems operate synergistically to support evidence-informed occupational health policies and workplace safety programs, mostly in high-income countries [26]. However, comparable surveillance infrastructures remain underdeveloped in many Sub-Saharan African countries. Strengthening occupational hazard incident reporting systems while progressively integrating them into broader occupational health surveillance frameworks is a long-term strategy for improving healthcare worker safety across the region [27].

This scoping review will provide a comprehensive overview of occupational hazard incident reporting systems among health workers in Sub-Saharan Africa. By mapping existing evidence, the review will shed light on how occupational incidents are reported, managed, and used for learning within health systems in the region. The findings are expected to reveal significant variability in the availability and functionality of reporting systems. Additionally, the review is set to identify barriers, including underreporting, weak feedback mechanisms, and limited integration into national health information systems. Identifying facilitators and best practices will be crucial for informing the design of context-appropriate interventions [17]. Future studies should also investigate organisational determinants of reporting behaviour and reporting culture within the region. More evidence could be generated concerning integration with digital health technologies and the impact of reporting systems on occupational safety outcomes. Such evidence will be essential for informing context-specific policies and strengthening occupational health systems capable of protecting healthcare workers throughout the region.

## Supporting information

S1 File A PRISMA ScR protocol checklist developed alongside this manuscript. (DOCX)

## Abbreviations

JBI: Joanna Briggs Institute
MeSH: Medical Subject Headings
PRISMA-ScR: Preferred Reporting Items for Systematic Reviews and Meta-Analyses extension for Scoping Reviews
SSA: sub-Saharan Africa
WHO: World Health Organisation.
