# Supplementary material for: Occupational hazard incident reporting systems in health facilities across sub-Saharan Africa: A scoping review protocol
Source: PLoS One. 2026 Oct 6;21(10):e0360189. doi: 10.1371/journal.pone.0360189 (PMC13641383; doi:10.1371/journal.pone.0360189)
Supplement: Supplementary file 1 [file pone.0360189.s003.pdf]

Dear editor, thank you so much for the opportunity to review our paper and have it improved. We have addressed all the reviewers comments and are resubmitting it for your consideration as described in this point-by-point response

Reviewer #1

Comment

I think this is an important piece of work related to occupational health and safety of healthcare workers. I have a few points to refine the work as listed below:

Introduction section: please also mention the category of psychosocial hazards for healthcare workers as that is very important considering current scenario. These workers face burnout due to increasing demands and are also exposed to violence and aggression from patients and their attendants - these are all forms of occupational hazard and should be preferably included in Incident Monitoring Systems.

Response

Dear reviewer, Thank you for this important observation. We agree that psychosocial hazards constitute a major occupational health challenge for healthcare workers and should be recognized alongside biological, chemical, physical and ergonomic hazards. Accordingly, we have expanded the Introduction to include psychosocial hazards such as occupational stress, burnout, workplace violence, bullying, verbal abuse, moral distress, and aggression from patients and visitors. We also highlight that modern Occupational Hazard Incident Reporting Systems (OHIRS) should ideally capture these incidents to enable comprehensive surveillance and prevention strategies.

Comment

Methods section: please elaborate further on data extraction (page 8) especially related to the Incident Reporting Systems themselves. What do you mean by "description of incident reporting system"? what exact variables will be used to extract information on the systems. Moreover, what variables will be useful to describe the limitations of these systems? A little more clarity is needed around these aspects.

Response

We appreciate this suggestion. The data extraction section has been substantially expanded to provide greater clarity on the characteristics of the Incident Reporting Systems to be extracted.

Comment

Discussion: please include a section on the limitations of Incident Reporting Systems. These systems are primarily 'reactive' (you need to wait for an incident to happen before taking any corrective measures) in nature and capture only the tip of the iceberg due to limited reporting. There are various other limitations that need to be mentioned.

Have a look at this article by Pham et al.: https://doi.org/10.4081/jphr.2013.e27

Response

We thank the reviewer for highlighting this important literature. The Discussion has been expanded to include a dedicated subsection discussing the inherent limitations of Incident Reporting Systems.

Comment

I would also encourage you to bring into the discussion the other parallel systems that may capture aspects of occupational hazards and exposures and are relevant for a comprehensive understanding and to have an idea about the scope of IRS. These include Occupational Safety and Health Monitoring systems and Occupational Health (disease and injury) Surveillance Systems. Although these systems may have limited - or I wonder, no presence at all - in Sub-Saharan Africa, it is important that you guide the reader to these broader approaches in your discussion and mention that in the longer run, such systems should be developed to address the broader issues of occupational health and safety of healthcare workers. for this purpose, have a look at the article here: Gaiotto, Emiliana Maria Grando1; Godoy Vieira, Aline1; Soares, Cássia Baldini1,2. Workers' occupational health surveillance systems in low- and middle-income countries: a scoping review protocol. JBI Evidence Synthesis 18(9):p 2098-2103, September 2020. | DOI: 10.11124/JBISRIR-D-19-00242

Response

We appreciate this insightful recommendation. We have broadened the Discussion to distinguish Occupational Hazard Incident Reporting Systems from broader Occupational Safety and Health (OSH) Monitoring Systems and Occupational Health Surveillance Systems.

Comment

Finally, mention something about the need of local research and scientific evidence on occupational health and safety and interventions / systems for countries in the region.

Response

Thank you for this suggestion. We have strengthened the concluding section of the Discussion by emphasizing the need for locally generated evidence to inform occupational health policies, implementation strategies, and context-specific interventions in Sub-Saharan Africa.

Reviewer #2:

Comment

Explicitly link the rationale to nursing education, clinical training, and nursing workforce safety.

Response

Thank you for this recommendation. The Introduction has been revised to explicitly discuss the implications of occupational hazard reporting systems for nursing education, clinical competency development, workplace safety culture, and protection of the nursing workforce. We highlight how robust reporting systems provide learning opportunities that inform safer clinical practice and support continuous professional development.

Comment

Include the full Boolean search strategy as a Supplementary File.

Response

The search strategy is already included within the manuscript.

Comment

Add AJOL and CINAHL to the database list, and specify grey literature sources.

Response

Thank you. AJOL, CINAHL, and grey literature sources are already included in the manuscript. The section reads as below: "A comprehensive search strategy shall be developed following consultation with a librarian. Various electronic databases, including PubMed/MEDLINE, Scopus, Web of Science, Embase, and CINAHL, will be used in the search strategy. Grey literature shall also be considered, and a search will be conducted across databases, including the World Health Organisation, the International Labour Organisation, Ministries of Health Websites, conference proceedings, and reports from theses and dissertation databases. The search will include studies published between 1990 and the date of the final search. No language restrictions will be applied, although studies not published in English will be translated where feasible. A combination of Medical Subject Headings (MeSH) and free-text terms will be used, including terms related to occupational hazards, incident reporting, surveillance systems, health workers, and Sub-Saharan Africa, as demonstrated in Table 1. Both controlled vocabularies, like MeSH and CINAHL Headings, and free-text keywords will be used. Boolean operators, OR and AND, shall be used to link the search strings."

Comment

Add a strong justification for omitting quality appraisal, or include a planned appraisal tool.

Response

Thank you for your recommendation. We have included a paragraph for quality appraisal within the manuscript.

Comment

Remove any actual "Results" or "Discussion of findings" to preserve the Protocol format; replace with a "Dissemination Plan" or "Protocol Strengths/Limitations."

Response

Thank you for that recommendation. The manuscript doesnot include discussion of findings section.

Comment

Upload the completed PRISMA-ScR checklist as a supplementary file.

Response

The updated PRISMA-ScR checklist has been uploaded.

Comment

Add the explicit statement exempting the protocol from ethical approval.

Response

Thank you for that recommendation. An ethical statement was included in the manuscript and read like this: "The study shall evaluate secondary information from published literature. Ethical approval has therefore not been sought to conduct this study. All authors consent to the publication of this protocol."

Comment

Ensure the abstract format matches PLOS ONE protocol requirements.

Response

We have evaluated PLOS ONE protocol requirements and have aligned the abstract accordingly.
