# Supplementary material for: Occupational hazard incident reporting systems in health facilities across sub-Saharan Africa: A scoping review protocol
Source: PLoS One. 2026 Oct 6;21(10):e0360189. doi: 10.1371/journal.pone.0360189 (PMC13641383; doi:10.1371/journal.pone.0360189)
Supplement: Peer review history

# Peer review: Decision Letter 0

**DOI:** 10.1371/journal.pone.0360189.r001

**Authors:** Sarah Jose

6 Jul 2026

Dear Dr. Innocent,

Thank you for submitting your manuscript to PLOS ONE. After careful consideration, we feel that it has merit but does not fully meet PLOS ONE’s publication criteria as it currently stands. Therefore, we invite you to submit a revised version of the manuscript that addresses the points raised during the review process.

Two reviewers have provided valuable feedback on your manuscript, which is included below. Please consider their suggestions and revise your manuscript accordingly, providing a point-by-point response to each comment upon resubmission. I note that you have included a PRISMA-ScR checklist, but please do update the page numbers as applicable following your revisions.

Please submit your revised manuscript by Aug 05 2026 11:59PM. If you will need more time than this to complete your revisions, please reply to this message or contact the journal office at plosone@plos.org. When you're ready to submit your revision, log on to https://www.editorialmanager.com/pone/ and select the 'Submissions Needing Revision' folder to locate your manuscript file.

- A letter that responds to each point raised by the academic editor and reviewer(s). You should upload this letter as a separate file labeled 'Response to Reviewers'.
- A marked-up copy of your manuscript that highlights changes made to the original version. You should upload this as a separate file labeled 'Revised Manuscript with Track Changes'.
- An unmarked version of your revised paper without tracked changes. You should upload this as a separate file labeled 'Manuscript'.

If you would like to make changes to your financial disclosure, please include your updated statement in your cover letter. Guidelines for resubmitting your figure files are available below the reviewer comments at the end of this letter.

If applicable, we recommend that you deposit your laboratory protocols in protocols.io to enhance the reproducibility of your results. Protocols.io assigns your protocol its own identifier (DOI) so that it can be cited independently in the future. For instructions see: https://journals.plos.org/plosone/s/submission-guidelines#loc-laboratory-protocols. Additionally, PLOS ONE offers an option for publishing peer-reviewed Lab Protocol articles, which describe protocols hosted on protocols.io. Read more information on sharing protocols at https://plos.org/protocols?utm_medium=editorial-email&utm_source=authorletters&utm_campaign=protocols.

As the corresponding author, your ORCID iD is verified in the submission system and will appear in the published article. PLOS supports the use of ORCID, and we encourage all coauthors to register for an ORCID iD and use it as well. Please encourage your coauthors to verify their ORCID iD within the submission system before final acceptance, as unverified ORCID iDs will not appear in the published article. *Only*  the individual author can complete the verification step; PLOS staff *cannot*  verify ORCID iDs on behalf of authors.

We look forward to receiving your revised manuscript.

Kind regards,

Sarah Jose, Ph.D.

Staff Editor

PLOS One

Journal Requirements:

When submitting your revision, we need you to address these additional requirements.

1. Please ensure that your manuscript meets PLOS ONE's style requirements, including those for file naming. The PLOS ONE style templates can be found at

https://journals.plos.org/plosone/s/file?id=wjVg/PLOSOne_formatting_sample_main_body.pdf and

https://journals.plos.org/plosone/s/file?id=ba62/PLOSOne_formatting_sample_title_authors_affiliations.pdf.

2. Please include captions for your Supporting Information files at the end of your manuscript, and update any in-text citations to match accordingly. Please see our Supporting Information guidelines for more information: http://journals.plos.org/plosone/s/supporting-information.

3. If the reviewer comments include a recommendation to cite specific previously published works, please review and evaluate these publications to determine whether they are relevant and should be cited. There is no requirement to cite these works unless the editor has indicated otherwise.

[Note: HTML markup is below. Please do not edit.]

Reviewers' comments:

Reviewer's Responses to Questions

**Comments to the Author**

1. Does the manuscript provide a valid rationale for the proposed study, with clearly identified and justified research questions?

Reviewer #1: Yes

Reviewer #2: Yes

**********

2. Is the protocol technically sound and planned in a manner that will lead to a meaningful outcome and allow testing the stated hypotheses?

Reviewer #1: Yes

Reviewer #2: Yes

**********

3. Is the methodology feasible and described in sufficient detail to allow the work to be replicable?

Reviewer #1: Yes

Reviewer #2: Yes

**********

4. Have the authors described where all data underlying the findings will be made available when the study is complete?

The PLOS Data policy

Reviewer #1: Yes

Reviewer #2: Yes

**********

5. Is the manuscript presented in an intelligible fashion and written in standard English?

Reviewer #1: Yes

Reviewer #2: Yes

**********

Please use the space provided to explain your answers to the questions above and, if applicable, provide comments about issues authors must address before this protocol can be accepted for publication. You may also include additional comments for the author, including concerns about research or publication ethics.

You may also provide optional suggestions and comments to authors that they might find helpful in planning their study.

Reviewer #1: I think this is an important piece of work related to occupational health and safety of healthcare workers. I have a few points to refine the work as listed below:

Introduction section: please also mention the category of psychosocial hazards for healthcare workers as that is very important considering current scenario. These workers face burnout due to increasing demands and are also exposed to violence and aggression from patients and their attendants - these are all forms of occupational hazard and should be preferably included in Incident Monitoring Systems.

Methods section: please elaborate further on data extraction (page 8) especially related to the Incident Reporting Systems themselves. What do you mean by "description of incident reporting system"? what exact variables will be used to extract information on the systems. Moreover, what variables will be useful to describe the limitations of these systems? A little more clarity is needed around these aspects.

Discussion: please include a section on the limitations of Incident Reporting Systems. These systems are primarily 'reactive' (you need to wait for an incident to happen before taking any corrective measures) in nature and capture only the tip of the iceberg due to limited reporting. There are various other limitations that need to be mentioned.

Have a look at this article by Pham et al.: https://doi.org/10.4081/jphr.2013.e27

I would also encourage you to bring into the discussion the other parallel systems that may capture aspects of occupational hazards and exposures and are relevant for a comprehensive understanding and to have an idea about the scope of IRS. These include Occupational Safety and Health Monitoring systems and Occupational Health (disease and injury) Surveillance Systems. Although these systems may have limited - or I wonder, no presence at all - in Sub-Saharan Africa, it is important that you guide the reader to these broader approaches in your discussion and mention that in the longer run, such systems should be developed to address the broader issues of occupational health and safety of healthcare workers.

for this purpose, have a look at the article here: Gaiotto, Emiliana Maria Grando1; Godoy Vieira, Aline1; Soares, Cássia Baldini1,2. Workers’ occupational health surveillance systems in low- and middle-income countries: a scoping review protocol. JBI Evidence Synthesis 18(9):p 2098-2103, September 2020. | DOI: 10.11124/JBISRIR-D-19-00242

Finally, mention something about the need of local research and scientific evidence on occupational health and safety and interventions / systems for countries in the region.

Reviewer #2: Explicitly link the rationale to nursing education, clinical training, and nursing workforce safety.

Include the full Boolean search strategy as a Supplementary File.

Add AJOL and CINAHL to the database list, and specify grey literature sources.

Add a strong justification for omitting quality appraisal, or include a planned appraisal tool.

Remove any actual "Results" or "Discussion of findings" to preserve the Protocol format; replace with a "Dissemination Plan" or "Protocol Strengths/Limitations."

Upload the completed PRISMA-ScR checklist as a supplementary file.

Add the explicit statement exempting the protocol from ethical approval.

Ensure the abstract format matches PLOS ONE protocol requirements.

**********

what does this mean?). If published, this will include your full peer review and any attached files.

If you choose “no”, your identity will remain anonymous but your review may still be made public.

**Do you want your identity to be public for this peer review?**  For information about this choice, including consent withdrawal, please see our Privacy Policy

Reviewer #1: **Yes:**  Asaad Ahmed Nafees

Reviewer #2: No

**********

[NOTE: If reviewer comments were submitted as an attachment file, they will be attached to this email and accessible via the submission site. Please log into your account, locate the manuscript record, and check for the action link "View Attachments". If this link does not appear, there are no attachment files.]

To ensure your figures meet our technical requirements, please review our figure guidelines: https://journals.plos.org/plosone/s/figures

You may also use PLOS’s free figure tool, NAAS, to help you prepare publication quality figures: https://journals.plos.org/plosone/s/figures#loc-tools-for-figure-preparation.

NAAS will assess whether your figures meet our technical requirements by comparing each figure against our figure specifications.

# Author comment: Author response to Decision Letter 1

**DOI:** 10.1371/journal.pone.0360189.r002

9 Jul 2026

Dear editor, thank you so much for the opportunity to review our paper and have it improved. We have addressed all the reviewers comments and are resubmitting it for your consideration as described in this point-by-point response

Reviewer #1

Comment

I think this is an important piece of work related to occupational health and safety of healthcare workers. I have a few points to refine the work as listed below:

Introduction section: please also mention the category of psychosocial hazards for healthcare workers as that is very important considering current scenario. These workers face burnout due to increasing demands and are also exposed to violence and aggression from patients and their attendants - these are all forms of occupational hazard and should be preferably included in Incident Monitoring Systems.

Response

Dear reviewer, Thank you for this important observation. We agree that psychosocial hazards constitute a major occupational health challenge for healthcare workers and should be recognized alongside biological, chemical, physical and ergonomic hazards. Accordingly, we have expanded the Introduction to include psychosocial hazards such as occupational stress, burnout, workplace violence, bullying, verbal abuse, moral distress, and aggression from patients and visitors. We also highlight that modern Occupational Hazard Incident Reporting Systems (OHIRS) should ideally capture these incidents to enable comprehensive surveillance and prevention strategies.

Comment

Methods section: please elaborate further on data extraction (page 8) especially related to the Incident Reporting Systems themselves. What do you mean by "description of incident reporting system"? what exact variables will be used to extract information on the systems. Moreover, what variables will be useful to describe the limitations of these systems? A little more clarity is needed around these aspects.

Response

We appreciate this suggestion. The data extraction section has been substantially expanded to provide greater clarity on the characteristics of the Incident Reporting Systems to be extracted.

Comment

Discussion: please include a section on the limitations of Incident Reporting Systems. These systems are primarily 'reactive' (you need to wait for an incident to happen before taking any corrective measures) in nature and capture only the tip of the iceberg due to limited reporting. There are various other limitations that need to be mentioned.

Have a look at this article by Pham et al.: https://doi.org/10.4081/jphr.2013.e27

Response

We thank the reviewer for highlighting this important literature. The Discussion has been expanded to include a dedicated subsection discussing the inherent limitations of Incident Reporting Systems.

Comment

I would also encourage you to bring into the discussion the other parallel systems that may capture aspects of occupational hazards and exposures and are relevant for a comprehensive understanding and to have an idea about the scope of IRS. These include Occupational Safety and Health Monitoring systems and Occupational Health (disease and injury) Surveillance Systems. Although these systems may have limited - or I wonder, no presence at all - in Sub-Saharan Africa, it is important that you guide the reader to these broader approaches in your discussion and mention that in the longer run, such systems should be developed to address the broader issues of occupational health and safety of healthcare workers. for this purpose, have a look at the article here: Gaiotto, Emiliana Maria Grando1; Godoy Vieira, Aline1; Soares, Cássia Baldini1,2. Workers’ occupational health surveillance systems in low- and middle-income countries: a scoping review protocol. JBI Evidence Synthesis 18(9):p 2098-2103, September 2020. | DOI: 10.11124/JBISRIR-D-19-00242

Response

We appreciate this insightful recommendation. We have broadened the Discussion to distinguish Occupational Hazard Incident Reporting Systems from broader Occupational Safety and Health (OSH) Monitoring Systems and Occupational Health Surveillance Systems.

Comment

Finally, mention something about the need of local research and scientific evidence on occupational health and safety and interventions / systems for countries in the region.

Response

Thank you for this suggestion. We have strengthened the concluding section of the Discussion by emphasizing the need for locally generated evidence to inform occupational health policies, implementation strategies, and context-specific interventions in Sub-Saharan Africa.

Reviewer #2:

Comment

Explicitly link the rationale to nursing education, clinical training, and nursing workforce safety.

Response

Thank you for this recommendation. The Introduction has been revised to explicitly discuss the implications of occupational hazard reporting systems for nursing education, clinical competency development, workplace safety culture, and protection of the nursing workforce. We highlight how robust reporting systems provide learning opportunities that inform safer clinical practice and support continuous professional development.

Comment

Include the full Boolean search strategy as a Supplementary File.

Response

The search strategy is already included within the manuscript.

Comment

Add AJOL and CINAHL to the database list, and specify grey literature sources.

Response

Thank you. AJOL, CINAHL, and grey literature sources are already included in the manuscript. The section reads as below: "A comprehensive search strategy shall be developed following consultation with a librarian. Various electronic databases, including PubMed/MEDLINE, Scopus, Web of Science, Embase, and CINAHL, will be used in the search strategy. Grey literature shall also be considered, and a search will be conducted across databases, including the World Health Organisation, the International Labour Organisation, Ministries of Health Websites, conference proceedings, and reports from theses and dissertation databases. The search will include studies published between 1990 and the date of the final search. No language restrictions will be applied, although studies not published in English will be translated where feasible. A combination of Medical Subject Headings (MeSH) and free-text terms will be used, including terms related to occupational hazards, incident reporting, surveillance systems, health workers, and Sub-Saharan Africa, as demonstrated in Table 1. Both controlled vocabularies, like MeSH and CINAHL Headings, and free-text keywords will be used. Boolean operators, OR and AND, shall be used to link the search strings."

Comment

Add a strong justification for omitting quality appraisal, or include a planned appraisal tool.

Response

Thank you for your recommendation. We have included a paragraph for quality appraisal within the manuscript.

Comment

Remove any actual "Results" or "Discussion of findings" to preserve the Protocol format; replace with a "Dissemination Plan" or "Protocol Strengths/Limitations."

Response

Thank you for that recommendation. The manuscript doesnot include discussion of findings section.

Comment

Upload the completed PRISMA-ScR checklist as a supplementary file.

Response

The updated PRISMA-ScR checklist has been uploaded.

Comment

Add the explicit statement exempting the protocol from ethical approval.

Response

Thank you for that recommendation. An ethical statement was included in the manuscript and read like this: "The study shall evaluate secondary information from published literature. Ethical approval has therefore not been sought to conduct this study. All authors consent to the publication of this protocol."

Comment

Ensure the abstract format matches PLOS ONE protocol requirements.

Response

We have evaluated PLOS ONE protocol requirements and have aligned the abstract accordingly.

Attachment Submitted filename: Response to reviewers.pdf

# Peer review: Decision Letter 1

**DOI:** 10.1371/journal.pone.0360189.r003

**Authors:** Laxit Bhatt

23 Sep 2026

Occupational Hazard Incident Reporting Systems in Health Facilities across Sub-Saharan Africa: A scoping Review Protocol

PONE-D-26-22378R1

Dear Dr. Innocent,

We’re pleased to inform you that your manuscript has been judged scientifically suitable for publication and will be formally accepted for publication once it meets all outstanding technical requirements.

Within one week, you’ll receive an e-mail detailing the required amendments. When these have been addressed, you’ll receive a formal acceptance letter and your manuscript will be scheduled for publication.

An invoice will be generated when your article is formally accepted. Please note, if your institution has a publishing partnership with PLOS and your article meets the relevant criteria, all or part of your publication costs will be covered. Please make sure your user information is up-to-date by logging into Editorial Manager at Editorial Manager® and clicking the ‘Update My Information' link at the top of the page. For questions related to billing, please contact billing support.

If your institution or institutions have a press office, please notify them about your upcoming paper to help maximize its impact. If they’ll be preparing press materials, please inform our press team as soon as possible -- no later than 48 hours after receiving the formal acceptance. Your manuscript will remain under strict press embargo until 2 pm Eastern Time on the date of publication. For more information, please contact onepress@plos.org.

Kind regards,

Laxit K Bhatt

Academic Editor

PLOS One

Additional Editor Comments (optional):

Reviewers' comments:

Reviewer's Responses to Questions

**Comments to the Author**

1. Does the manuscript provide a valid rationale for the proposed study, with clearly identified and justified research questions?

Reviewer #2: Yes

Reviewer #3: Yes

**********

2. Is the protocol technically sound and planned in a manner that will lead to a meaningful outcome and allow testing the stated hypotheses?

Reviewer #2: Yes

Reviewer #3: Yes

**********

3. Is the methodology feasible and described in sufficient detail to allow the work to be replicable?

Reviewer #2: Yes

Reviewer #3: Yes

**********

4. Have the authors described where all data underlying the findings will be made available when the study is complete?

The PLOS Data policy

Reviewer #2: Yes

Reviewer #3: Yes

**********

5. Is the manuscript presented in an intelligible fashion and written in standard English?

Reviewer #2: Yes

Reviewer #3: Yes

**********

Please use the space provided to explain your answers to the questions above and, if applicable, provide comments about issues authors must address before this protocol can be accepted for publication. You may also include additional comments for the author, including concerns about research or publication ethics.

You may also provide optional suggestions and comments to authors that they might find helpful in planning their study.

Reviewer #2: Overall Assessment

The authors have done an excellent job addressing the core concerns raised in the previous round of review. The inclusion of psychosocial hazards, the detailed expansion of the data extraction variables, the addition of the critical appraisal plan, and the explicit statement regarding ethical approval have significantly strengthened the protocol. The methodology is now robust, well-articulated, and perfectly aligned with JBI scoping review guidelines.

The manuscript makes a valuable contribution to the field of occupational health and safety in Sub-Saharan Africa, and I am pleased to see it in its current form.

Reviewer #3: The review excellently captured the scoping review of incident reporting in health facilities. The methodology were robust and were clearly explained.

**********

what does this mean?). If published, this will include your full peer review and any attached files.

If you choose “no”, your identity will remain anonymous but your review may still be made public.

**Do you want your identity to be public for this peer review?**  For information about this choice, including consent withdrawal, please see our Privacy Policy

Reviewer #2: No

Reviewer #3: No

**********

# Editor report: Acceptance letter

**DOI:** 10.1371/journal.pone.0360189.r004

**Authors:** Laxit Bhatt

PONE-D-26-22378R1

PLOS One

Dear Dr. Ayesiga,

I'm pleased to inform you that your manuscript has been deemed suitable for publication in PLOS One. Congratulations! Your manuscript is now being handed over to our production team.

At this stage, our production department will prepare your paper for publication. This includes ensuring the following:

* All references, tables, and figures are properly cited

* All relevant supporting information is included in the manuscript submission,

* There are no issues that prevent the paper from being properly typeset

You will receive further instructions from the production team, including instructions on how to review your proof when it is ready. Please keep in mind that we are working through a large volume of accepted articles, so please give us a few days to review your paper and let you know the next and final steps.

Lastly, if your institution or institutions have a press office, please let them know about your upcoming paper now to help maximize its impact. If they'll be preparing press materials, please inform our press team within the next 48 hours. Your manuscript will remain under strict press embargo until 2 pm Eastern Time on the date of publication. For more information, please contact onepress@plos.org.

You will receive an invoice from PLOS for your publication fee after your manuscript has reached the completed accept phase. If you receive an email requesting payment before acceptance or for any other service, this may be a phishing scheme. Learn how to identify phishing emails and protect your accounts at https://explore.plos.org/phishing.

If we can help with anything else, please email us at customercare@plos.org.

Thank you for submitting your work to PLOS One and supporting open access.

Kind regards,

PLOS One Editorial Office Staff

on behalf of

Mr. Laxit K Bhatt

Academic Editor

PLOS One
